# Supplementary figures and images for: p63 cooperates with CTCF to modulate chromatin architecture in skin keratinocytes
Source: Epigenetics Chromatin. 2019 Jun 4;12:31. doi: 10.1186/s13072-019-0280-y (PMC6547520; doi:10.1186/s13072-019-0280-y)

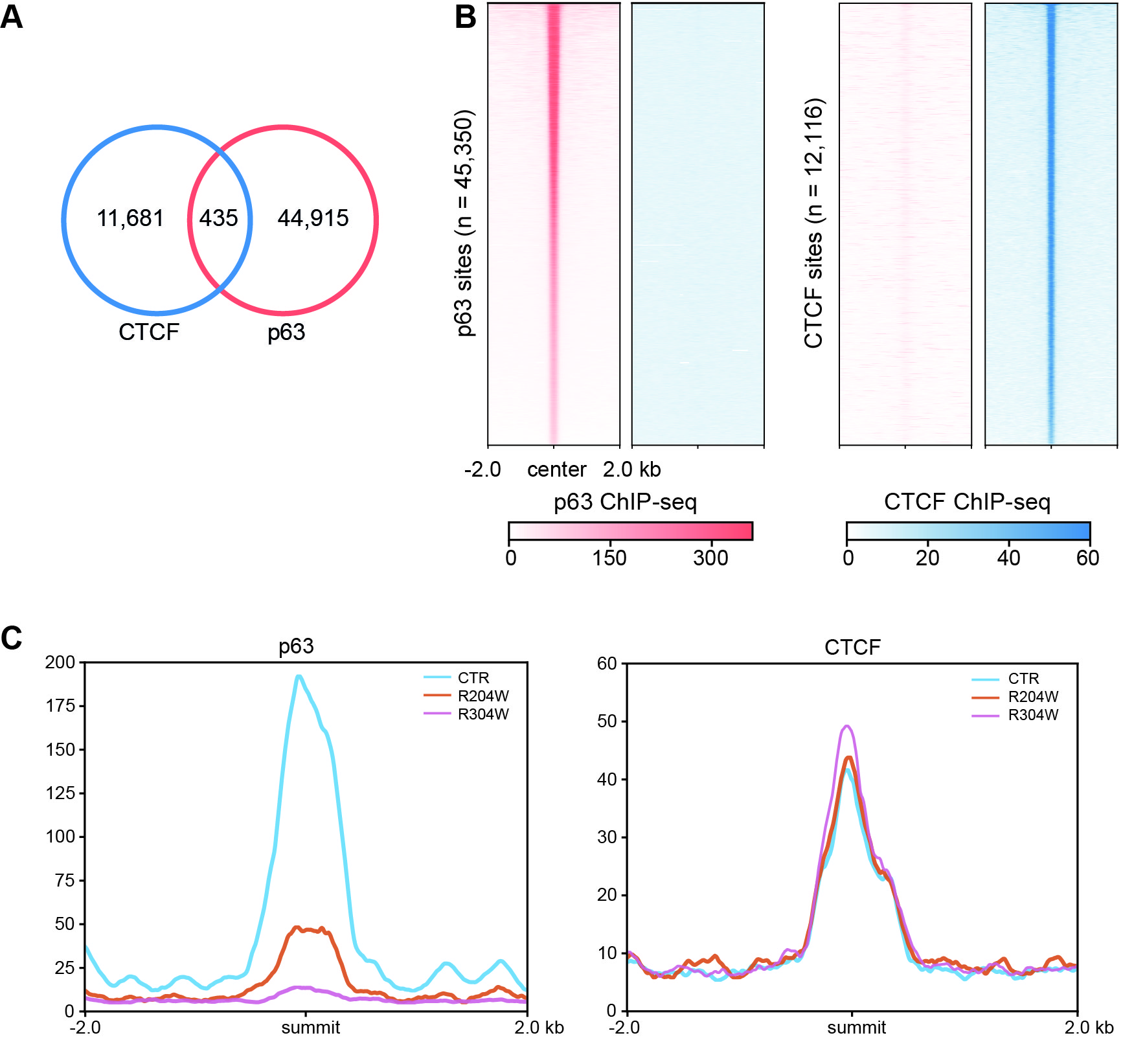

Supplement: Supplementary file 1 — Additional file 1: Figure S1. Few overlap between p63 and CTCF binding sites. A Overlapping of p63 and CTCF binding sites from ChIP-seq data. B Heatmaps showing the CTCF binding signal at all 45,350 p63 binding sites (left) and the p63 binding signal at all 12,116 CTCF binding sites (right). C Bandplots showing the p63 binding signal (left) and CTCF binding signal (right) at the 435 co-binding sites in both control and p63 mutant keratinocytes. [file 13072_2019_280_MOESM1_ESM.docx]

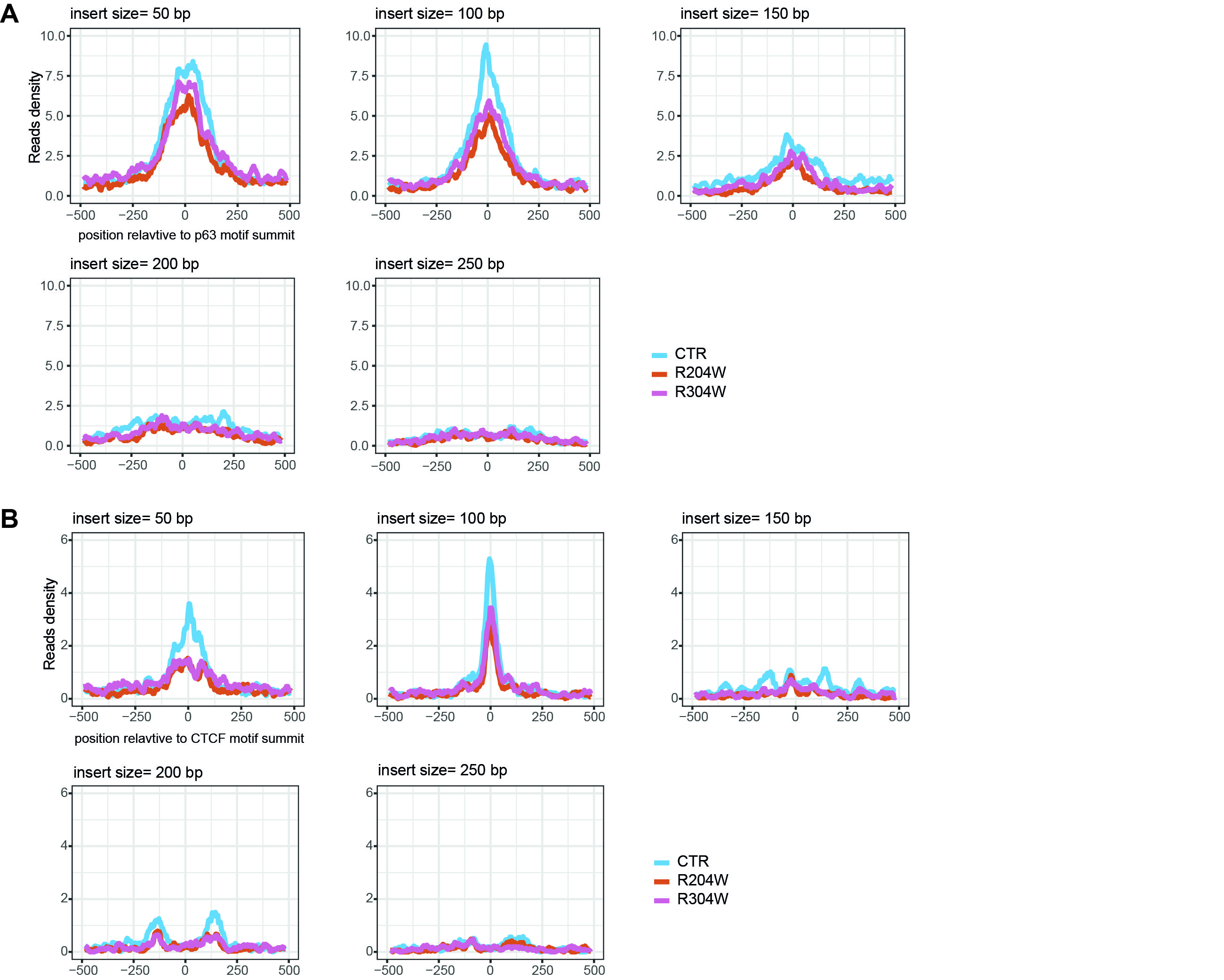

Supplement: Supplementary file 2 — Additional file 2: Figure S2. Deregulated nucleosome organization at p63-bound and CTCF-bound Ctr-OCRs in p63 mutant keratinocytes. A Reads density of ATAC-seq at p63-bound Ctr-OCRs with different insert sizes in both control and p63 mutant keratinocytes. B Reads density of ATAC-seq at CTCF-bound Ctr-OCRs with different insert sizes in both control and p63 mutant keratinocytes. [file 13072_2019_280_MOESM2_ESM.docx]

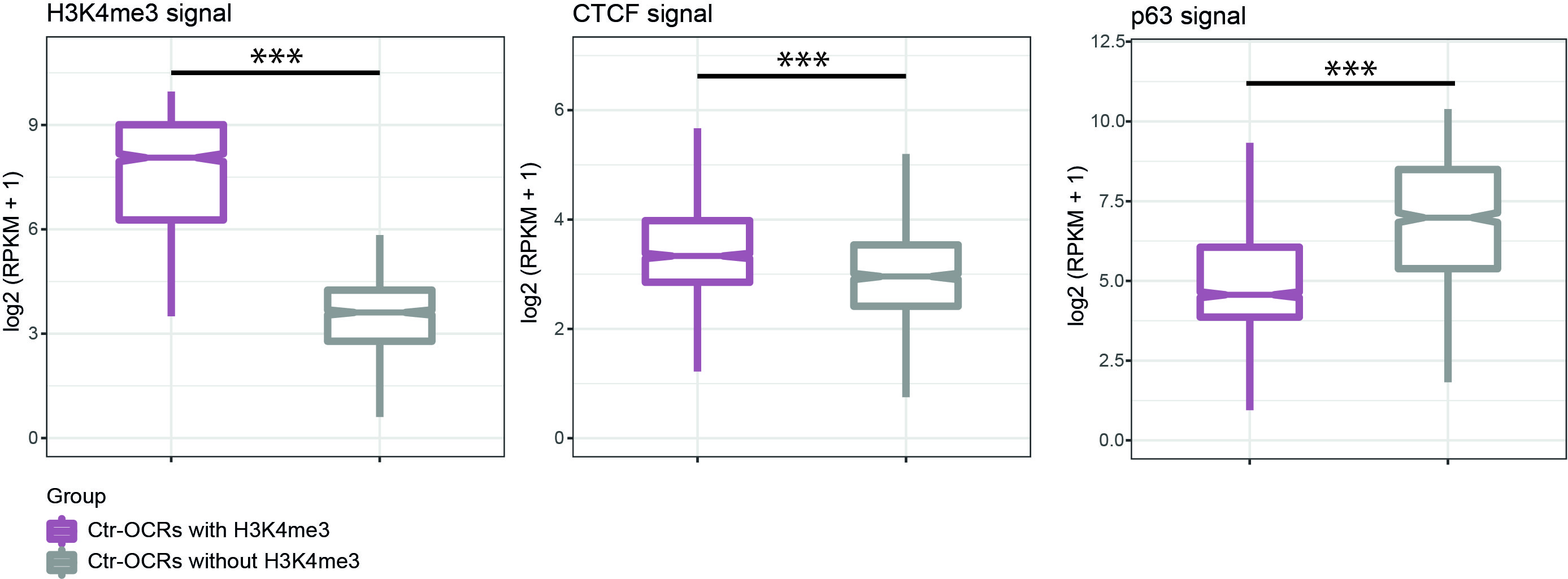

Supplement: Supplementary file 3 — Additional file 3: Figure S3. CTCF bound preferentially to Ctr-OCRs marked by H3K4me3. Ctr-OCRs are grouped into two categories according to H3K4me3 signal, Ctr-OCRs with H3K4me3 (purple) and Ctr-OCRs without H3K4me3 (gray). CTCF ChIP-seq showed higher signal in Ctr-OCRs with H3K4me3, while p63 ChIP-seq showed higher signal in Ctr-OCRs without H3K4me3 in control keratinocytes. ***, p < 0.001, Wilcoxon signed-rank test. [file 13072_2019_280_MOESM3_ESM.docx]
